# Personal mental health navigator: Harnessing the power of data, personal models, and health cybernetics to promote psychological well-being

**DOI:** 10.3389/fdgth.2022.933587

**Published:** 2022-09-22

**Authors:** Amir M. Rahmani, Jocelyn Lai, Salar Jafarlou, Iman Azimi, Asal Yunusova, Alex. P. Rivera, Sina Labbaf, Arman Anzanpour, Nikil Dutt, Ramesh Jain, Jessica L. Borelli

**Affiliations:** ^1^ Department of Computer Science, University of California, Irvine, CA, USA; ^2^ School of Nursing, University of California, Irvine, CA, USA; ^3^ Department of Psychological Science, University of California, Irvine, CA, USA; ^4^ Department of Computing, University of Turku, Turku, Finland; ^5^ Institute for Future Health, University of California, Irvine, CA, USA

**Keywords:** mental health, wearable internet-of-things, health cybernetics, personal health models, personal chronicle

## Abstract

Current digital mental healthcare solutions conventionally take on a reactive approach, requiring individuals to self-monitor and document existing symptoms. These solutions are unable to provide comprehensive, wrap-around, customized treatments that capture an individual’s holistic mental health model as it unfolds over time. Recognizing that each individual requires personally tailored mental health treatment, we introduce the notion of Personalized Mental Health Navigation (MHN): a cybernetic goal-based system that deploys a continuous loop of monitoring, estimation, and guidance to steer the individual towards mental flourishing. We present the core components of MHN that are premised on the importance of addressing an individual’s personal mental health state. Moreover, we provide an overview of the existing physical health navigation systems and highlight the requirements and challenges of deploying the navigational approach to the mental health domain.

## 1. Introduction

Mental health is an important factor in determining an individual’s quality of life. The current mental healthcare system typically deploys an acute, symptom-focused, reactive approach to address patient well-being as opposed to adopting a preventative approach that seeks to prevent illness from developing. One major drawback of this system is its passive approach to mental health. Furthermore, the traditional psychotherapy model of treatment is often premised on the notion that the individual is expected to be an accurate reporter of their symptoms and health. In many cases, individuals only become conscious of their issues once their conditions become severe or reach a point where they perceive a need for the issue to be addressed (1, 2). The field of mental health has increasingly recognized the inherent flaws in this methodology, where psychological states are not only subjective and culturally constrained (3–5), but that there are also reasons to be concerned about this method of reporting, such as the fact that clients often have poor memory and insight into the cause of their behavior (6, 7).

Recent shifts in health models—such as the inclusion of mental health providers within primary care settings (8, 9)—and improvements in technology offer complementary approaches to addressing these limitations focused on acute and symptom-focused approaches, by incorporating consideration of an individual’s unique lifestyle and exposome in the service of offering personalized interventions. The field of medicine has also long adopted the biopsychosocial model as a holistic approach towards understanding health and care. The biopsychosocial model considers both external (i.e., sociocultural) and internal factors (i.e., biology and psychology) that may impact health (10–13). External factors encompass a wide-range of contexts, including but not limited to family, community, environment, and larger societal structures. Dynamic interactions across these factors may relate to fluctuations in health across a continuum of well-being and quality of life.

Furthermore, with advances in ubiquitous and wearable sensing, it is possible to more objectively monitor such fluctuations in an individual’s position on the mental health continuum, offering tremendous opportunities and greater insight to better understand an individual’s needs outside their own self-reported experiences (14, 15) and help navigate them towards a healthy state of mind through personalized estimations and intervention. Depending on the severity and type of intervention, such services can be provided through a therapist-in-a-loop model or autonomously using smart guidance systems.

In this perspective paper, we propose the notion of Personalized Mental Health Navigation (MHN) as a goal-based closed-loop guidance system with the potential to contribute to a more continuous, preventative, navigational paradigm of mental health care that leverages a holistic, personalized model of the individual. We first present our vision of a MHN system and provide a high-level overview of its core components. We then provide an overview of the existing physical health navigation systems and highlight the requirements and challenges of deploying the navigational approach to the mental health domain. We also briefly discuss the existing monitoring and guidance methods that can be leveraged to realized the notion of MNH.

## 2. The personalized mental health navigator model

A navigational approach to health (16) can best be illustrated with a simple metaphor: imagine using a route navigator software (e.g., Google Maps) to navigate you from your current location to your desired destination. The route navigator software constantly monitors your location using GPS to estimate your current state on the map. The navigator then identifies the most efficient route (in part, based on your preference) and gives you step-by-step guidance on how to reach your destination. If you make a wrong turn, decide to make a stop, or encounter traffic, the navigator will promptly re-calibrate based on your decision or the external environment and adjust accordingly. The route navigator is an operational example of a cybernetic feedback control system (17).

**Figure 1:**
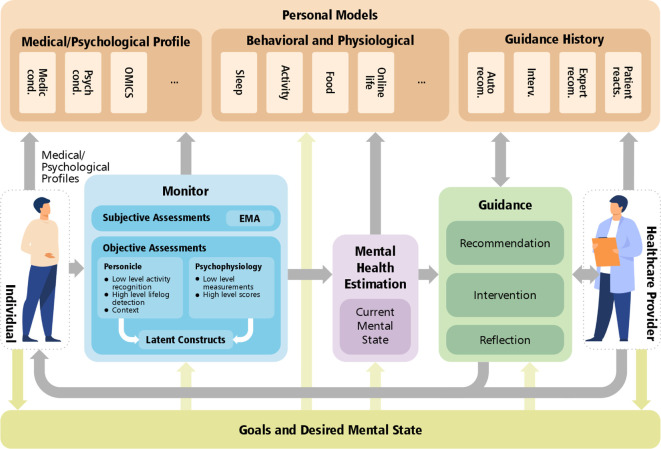
The personalized Mental Health Navigator (MHN) system enabled by a goal-based closed-loop guidance.

Inspired by such route navigators, we propose a personalized MHN system to plan and direct the route from the user’s current mental health state to the desired state. The MHN system adopts a cybernetics approach that allows for a continuous loop of measurement, monitoring, estimation, guidance (enabled by personal models), and influence to maintain and support a person’s mental health. The science of cybernetics is centered around setting *Goals* and devising action sequences to accomplish and maintain those goals in the presence of disturbances: e.g., an individual’s life challenges. In this perspective article, we propose a reference architecture for such systems that includes five major modules integrated in a cybernetics structure: (i) Goals and the desired mental state, (ii) Monitor, (iii) Mental Health Estimation, (iv) Personal Models, and (v) Guidance. A view of the system—including the modules and their interactions—is illustrated in Figure 1. In the sections that follow, we describe the modules in detail.

### 2.1. Goals and the desired mental state

*Goals and desired mental state* are the core of the MHN system. A personalized MHN system aims to capture, store, and keep track of the desired mental states and goals, which interact with the other modules. Mental state is a high-dimensional space referring to cognitions (thoughts and beliefs) and emotions (feelings and moods) that can comprise an individual’s experience. Individuals are motivated by conscious and unconscious goals that are then reflected in changes within their physical and mental states (18). The goals and desired mental states can consist of basic needs (e.g., sleep) or more complex goals (e.g., running for government to enact change) that altogether may play a role in the individual’s health and well-being. Events elicit a response within the individual that then leads to behaviors based on how the individual appraises the events (19). More broadly, individuals are motivated to seek enjoyable hedonic states through pursuing certain activities (e.g., by spending time with loved ones) or engage in behaviors that expose the individual to challenge or stress (e.g., studying for an important exam) if they recognize that it will benefit their long-term goals. In the context of mental health, life challenges or barriers to goal attainment can be considered as disturbances.

Goal setting is not a fixed state but can include a collaborative process between an individual and a healthcare provider that progresses in order to reach a goal consensus. An individual often seeks mental health services to address a specific mental health concern or self-improvement need (e.g., identified by the self). This goal might be nonspecific or ill-defined (e.g., “I want to feel better about myself or my life”), so the provider seeks to understand this need or goal better through an information gathering process. This process often includes understanding more about the context and timing in which the individual experiences the undesired or opposite state of the goal state. Doing so requires a holistic and comprehensive data acquisition procedure (i.e., the Monitor module). Through the MHN system, the same individual can monitor their own physical and psychological profiles through available tools that allow them to better understand, reflect, and initiate intervention. Seeking intervention and guidance from a provider can also help the individual work through the current states in order to attain desired states.

In addition, the MHN system could be used in the absence of the individual being involved in ongoing therapy. We propose that the MHN can be used as a model of mental health prevention, so that the MHN system is continuously monitoring the mental state of the individual in order to assess whether they need just-in-time brief interventions from the healthcare provider or the MHN system (e.g., app-based interventions) in order to prevent the need for more intensive interventions, such as ongoing psychotherapy. This type of model for the MHN mimics a prevention model of mental health care that is integrated into daily living in much the same way as mental health professionals are now integrated into primary care settings (they are integrated into routine health care check ups and can provide on-the-spot interventions, typically lasting 5–10 min within an office visit, if needed, e.g., (20)).

### 2.2. Monitor

**Figure 2:**
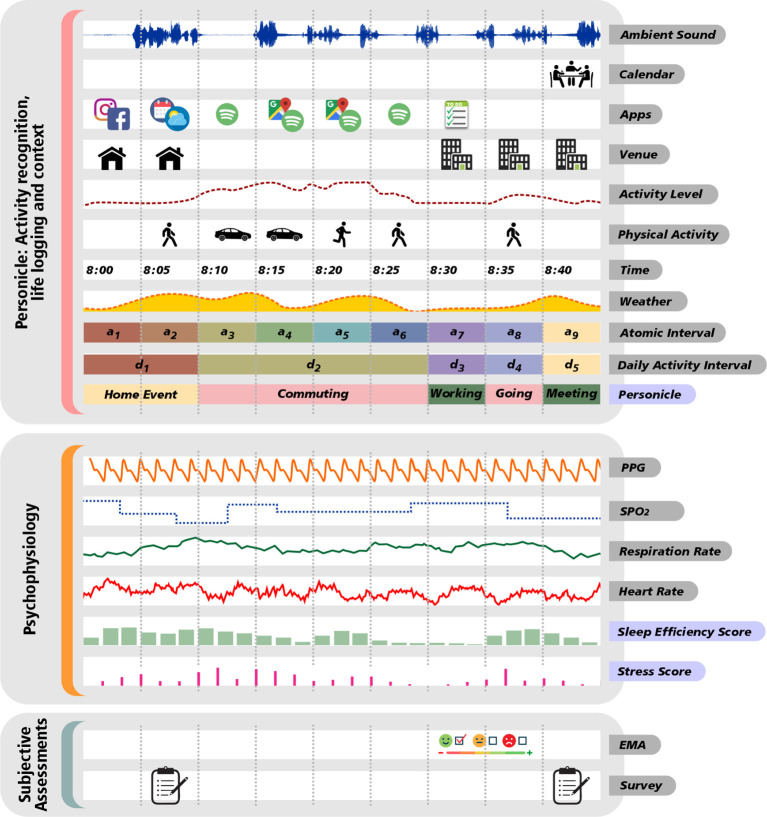
*Monitor* as the main subjective and objective data collection module.

*Monitor* is the main data acquisition module in the MHN model and serves as the gateway of subjective and objective information flow from the individual (see Figure 2). With the development of the Internet of Things (IoT) technology to capture experiences in real-time, researchers can monitor an individual’s subjective experiences, their behavioral and physiological experiences (21, 22), and contextual situation. In addition, this information can be exploited to further understand human phenomena that are difficult to capture (i.e., latent constructs such as loneliness or depression that involve subjective feelings as well as bodily and physiological sensations). This module can support multiple flows of information, described in the following.

#### 2.2.1. Ecological momentary assessments (EMA)

Ecological Momentary Assessments (EMA) is a longitudinal data collection technique, developed to capture in-the-moment subjective experiences as they occur (23). EMAs can offer a more thorough account of daily life and experiences at the individual-level through long-term monitoring. Recent literature calls for a more person-centered (i.e., within-person) approach, in which individualized modeling not only helps us understand variability in human behavior but also how psychopathology uniquely manifests for some individuals and ultimately improve interventions (24). EMA methodologies that include brief self-reported assessments provide subjective individual experiences that go hand-in-hand with objective experiences collected by technology.

#### 2.2.2. Personicle

The *Personicle* component (i.e., personal chronicle of daily events) objectively and automatically measures one’s life events to help the MHN system incrementally model the person based on multi-modal life logging measurements (see Figure 2). Contemporary lifelogging (i.e., capturing data of one’s daily events) and life event recognition technologies in the literature include situation specific recognition (e.g., smart home), computer vision-based recognition (e.g., surveillance), and sensor-based recognition (e.g., accelerometer). Such technologies enable capturing of low-level activities (e.g., step counts) but critically lack the ability to capture or infer important contextual and higher cognitive factors that enable assessment and prediction of a person’s lifestyle. In our preliminary work on building *Personicle* (Personal Chronicle) (25), we attempted to lay the foundation for a multi-modal life logging framework that i) integrates heterogeneous Body Area Network sensory data (e.g., activity, phone oriented context, and social interactions), ii) synchronizes these data streams, iii) recognizes high-level daily activity, and iv) builds a personal chronicle (*Personicle*) of daily activity for the individual (25). The *Personicle* model is intended to evolve using machine learning techniques applied to daily activities in the chronicle and relate them to biomedical or behavioral signals. The model enriches the other subjective and objective mental health related data of an individual by including context around them to be used later to build personalized models.

#### 2.2.3. Psychophysiological assessment

Along with lifelogging, the *Monitor* module collects users’ physiological data using ubiquitous devices, such as smart watches and rings. These devices are generally equipped to objectively monitor cardiac and autonomic nervous system activity through photoplethysmography (PPG) and electrodermal activity (EDA) methods. From PPG, one can extract HR (heart rate), respiration rate, and HRV (heart rate variability), i.e., a raw signal of sympathetic nervous system activity. Different signal processing methods could be applied on these extracted metrics, such as time difference of two consecutive peaks, frequency spectrum analysis, and non-linear assessments. In addition, EDA is a powerful method frequently used in smart wearable devices to measure skin conductance. Changes in gland activity cause abrupt variations in sweat level of the skin that affect the skin conductance. This metric is often used as an objective indicator of stress (26, 27).

Ample research links activation of physiological reactivity with mental health outcomes (28). For instance, elevations in sympathetic nervous system (SNS) activation, such as increases in heart rate (HR), pre-ejection period, or EDA have all been linked with mental health outcomes (21, 29). Moreover, greater decreases in parasympathetic nervous system (PNS) activation (HRV) have also been meaningfully associated with anxiety and depression (30). Similarly, objective bio-signal analysis to detect stress has been shown to be expandable to detect other psychological disorders, such as bipolar disorder that can significantly affect physiology (31). Sleep is another essential parameter that can be conveniently monitored using IoT devices (e.g., smart rings and watches).

#### 2.2.4. Modeling latent psychological constructs using multiple sources of data

Many human experiences and phenomena are often multi-dimensional, incorporating a person’s own perception and internal experiences as well as external factors. For these reasons, the *Monitor* module performs methods to fuse multi-modal assessments to capture latent psychological constructs. The module synthesizes several objective assessments to model, detect, and estimate larger psychological latent constructs that cannot be measured by a single parameter. This may include aspects, such as depression and loneliness. It should be noted that some of these constructs can be captured episodically using EMAs, however, it is not feasible to objectively and continuously measure such high-level latent constructs. Existing work in the field of Affective Computing have attempted to address this problem by building multi-modal predictive machine learning models to estimate individual’s high-level mental health (32–35). One example is calculating stress level using machine learning models by fusing several parameters extracted from biosignals, such Electrocardiogram, PPG, and EDA (36). Similarly, predictive models have been built to detect loneliness by fusing tens of physiological and behavioral metrics from HRV to location, phone engagement, and usage patterns (37)

### 2.3. Mental health estimation

The *Mental Health Estimation* module leverages the holistic high-dimensional data collected via *Monitor* to identify and estimate a person’s mental health. The module could help indicate clinical symptomatology and potential risk for a mental health disorder (33, 34). In the context of mental health estimation, different dimensions need to be considered to model and estimate, including emotional factors, behavioral traits, social factors, cultural factors, linguistic factors, cognitive state, biological markers (38), interpersonal relations, and, neurological state. While a large body of literature has investigated each of these dimensions often in isolation (38), the field could expand to estimate an individual’s mental health state in a holistic way, considering a multitude of factors and life experiences beyond passive physiological sensing. The objective of this module is to process different modalities to properly estimate mental health variables in different dimensions.

### 2.4. Personal models

The *Personal Models* module is conceptualized as building models of physiological, psychological, and behavioral patterns for each individual to personalize the monitoring, estimation, and guidance provided within the system. Such personalization services have already been developed for platforms, such as Netflix, YouTube, and Amazon wherein individualized algorithms are used and created for each client to deliver advertisements and services that cater to their interests and preferences.

In the case of the MHN system, a wide variety of data is collected for an individual through monitoring. Longitudinal data are leveraged to extract the relations among all measurements related to mental health. In addition to continuous data collection, a set of historical and demographic information of a patient’s mental and medical conditions are gathered through surveys and other medical documents. Demographic and medical history can be used, by the MHN system, as context to determine a more accurate mental state of the individual. Besides the data generated by different modules in MHN (i.e., *Monitor*, *Mental Health Estimation*, and *Guidance*), this module is ideally most effective when leveraging a range of other personal data, including prior mental/medical conditions experienced by the individual.

### 2.5. Guidance

The *Guidance* module in MHN represents the stage, in which users (i.e., individuals and healthcare providers) can synthesize information from the monitoring and estimation stages and determine whether they align with their goals and mental states. Individuals can *reflect* on their goals and progress and consider adjustments to their own behaviors. They can also seek the *recommendations* of the AI-based recommendation systems or a healthcare provider. The individual at this stage can opt to *intervene* to maintain, add, or revise goals in order to attain their desired mental states. Furthermore, individuals can increase their own self-efficacy by engaging in self-management behaviors to maintain their health.

Based on the level of detailed information that encompasses the monitoring stage (e.g., subjective, contextual information, and physiological fluctuations), a provider can offer professional recommendations or assist the individual in processing this information. The provider can support the individual in developing self-efficacy in the engagement of health behavior changes, and prescribe treatment when necessary. Once a plan of action or intervention is initiated from either with the AI-recommender system or from the help of a provider, the *Guidance* module then returns to the *Monitor* to continually observe the individual’s experiences in the process of treatment and changes in behavior.

## 3. State-of-the-art navigation systems for physical health

The goal-oriented navigation approach has recently entered the field of healthcare to provide iterative feedback and guidance with respect to the user’s current health status. It has been shown that such navigational systems can provide preventive intervention resulting in individuals’ lifestyle change and proactive care. For example, Nag et al. (39) propose a cardiovascular physical health navigation system in which the users are guided to improve their cardiac health state. Their proposed system—enabled by wearable and mobile sensing—continuously monitors individuals’ cardiovascular health status by collecting parameters, such as heart rate, VO2max, and sleep level. Then, the system calculates the risk of Atherosclerotic Cardiovascular Decease (ASCVD) and subsequently provides daily guidance to help the individuals reach their health goal. Navigation systems have been also introduced for diabetic patients. For instance, DiaNavi (i.e., Diabetes Navigator) (40, 41) is a lifestyle guidance system proposed for patients with Type II diabetes with the aim of continuously monitoring blood sugar and mitigating drug dependency. The system captures nutritional parameters, calories out, blood glucose level, etc. at a periodic interval. It, then, builds health models to estimate the variations in the health parameters and generate step-by-step recommendations. In another study, Pandey et al. (42) present a food preference modelling approach to generate food recommendations based on aspects of nutritional information, taste preferences, and contextual and social information.

These recently developed navigation systems for physical health are promising in their attempts to shift the healthcare paradigm. We believe the field of digital mental health can also embrace this paradigm shift. However, the deployment of this model in the mental health domain is not a trivial task. Challenges such as the following remain: (i) subjectivity of many mental health measurements, (ii) quantification of the current mental health state, (iii) high-dimensionality of the mental health space, (iv) the need for the receipt of step-by-step guidance for behavior change, and (v) the overwhelming confounding variables in everyday settings. These questions need to be further investigated to realize the full potential of the MHN model. However, as can be seen in the next section, there exist several efforts aiming to address some of these challenges (even though in isolation), for example, to objectively monitor mental health and provide guidance in everyday settings.

## 4. Ongoing efforts towards mental health monitoring and guidance

The majority of ongoing efforts in the digital mental health domain fall under (i) monitoring (i.e., assessment) or (ii) guidance (i.e., intervention/recommendation). Some of the works under the guidance follow the idea of just-in-time adaptive intervention (JITAI) (43) which often comes with near real-time assessments. In this section, we present some examples of the ongoing efforts in these spaces. It should be noted that this is not an extensive review of known efforts.

A plethora of monitoring systems have been proposed to track users’ mental health through self-report questionnaire or sensors. Smartphones and smart wearables allow individuals to self-monitor or track their own health and lifestyle behaviors remotely (44). For instance, Mozos et al. (45) propose a wearable-based approach for automatic stress detection, using EDA, PPG, speech, body movement, and proximity to other people. In another study, the correlation between late-life depression and physical activity has been assessed using an activity monitor (46). These monitoring systems mostly investigate relationships between different mental health-related factors or detect mental conditions including stress and anxiety (47, 48). However, they do not close the loop by providing feedback or recommendations for the users.

In contrast, guidance or recommendation systems have been introduced to provide interventions for individuals. For example, Purple Robot (49) is a comprehensive modular framework that uses both self-report and photo sensor data to assess and deploy behavioral intervention technologies. As part of Purple Robot, Mobilyze (50) was proposed as an extension intended to leverage mobile context sensing for depression intervention. Another example is the product, Ilumivu (51), which offers smart wearable integration and an advanced ruled-based intervention triggering system connecting patients and clinicians. Many of the existing implementations of such frameworks have yet to fully incorporate smart wearable technologies (i.e., watches/rings). While the current state of the field showcases evidence of progress in properly collecting and modeling user data, they lack a concrete notion of mental health state and navigation as an iterative (step-by-step) process. In the conventional guidance approaches, understanding individual differences is a major barrier to achieving a comprehensive mental health navigation service.

## 5. Conclusions and future work

In this paper, we proposed the notion of Personalized Mental Health Navigation (MHN) as a goal-based cybernetics system allowing for a continuous loop of monitor, estimation, guidance, and influence to monitor and support an individual’s desired mental health. The MHN system included multiple modules to incorporate a personalized model towards addressing mental health. The MHN offered one vision of how technology supports mental health, wherein providers could have a round-the-clock inside view into their clients’ lives, rendering it so that in-session interactions and recommendations can be supported by knowledge gleaned from out-of-session data points. We further discussed the state-of-the-art navigation systems proposed for physical health. As a further next step, extensive research is required to also assess non-technical aspects of MHN, such as preventing the system from potentially directing the user toward less adaptive health behaviors or legal/ethical concerns arising regarding privacy and security of data.
